# Physical fitness as an indicator of health status and its relationship to academic performance during the prepubertal period

**DOI:** 10.15171/hpp.2017.35

**Published:** 2017-09-26

**Authors:** Francisco Javier Fonseca del Pozo, Joaquín Valle Alonso, Manuel Vaquero Álvarez, Siobhan Orr, Francisco Jesús Llorente Cantarero

**Affiliations:** ^1^Department of Emergency and Critical Care Unit of Montoro, Maimónides Biomedical Research Institute of Córdoba, Córdoba, Spain; ^2^Department of Emergency Medicine, Royal Bournemouth Hospital, Bournemouth, Dorset, UK; ^3^Department of Family and Community Medicine, Primary Care Center, Jaen, Spain; ^4^Department of Community Research, Maimónides Biomedical Research Institute of Córdoba, Spain

**Keywords:** Academic performance, Physical fitness, Cognitive performance, Obesity

## Abstract

**Background:** Physical activity (PA) is considered one of the most important determinants of the health status in children, and predictor of morbidity/mortality in adults. The aim is to examine the relationship between physical fitness (PF), PA, obesity and academic performance (AP) in primary school children.

** Methods:** Cross-sectional studies including 91 primary school students, aged 9 to 12 years, from the province of Córdoba. Data was collected from April to June 2014. We measured PF using part of the EUROFIT fitness testing battery. The level of PA was measured as low or high PF and the level of obesity was measured using body mass index, waist circumference, percentage of fat mass, lean body mass, percentage of lean mass and basal metabolism. AP by scores on the second quarter was based on the total average of scores of basic subjects and other subjects, including physical education. Cognitive performance was assessed by the Spanish overall and factorial intelligence test.

**Results:** The results of AP were positively related to levels of PF. Students who achieve better PF score better in Maths, (P=0.019), Natural Sciences (P=0.024), Religion (P=0.018) and Physical Education (P<0.001). A direct association between maximal aerobic capacity with Mathematics (r=0.325, P=0.02), AP (r=0.349, P=0.001) and cognitive performance (CP)(r=0.312, P=0.003) was observed. There was also a direct association of better jump tests with higher AP (r=0.328, P=0.002).

**Conclusion:** The AP is associated with higher levels of fitness. Therefore, the education system should consider implementing curriculum strategies favouring the improvement of the PF, and therefore the health and AP of students.

## Introduction

In most developed countries, recent changes in children’s environments have resulted in the development of chronic diseases secondary to obesity, as described in a report by the World Health Organization (WHO).^1^ These changes include increased consumption of foods that are high in saturated fat and low in unrefined carbohydrates, i.e. “high-density energy”, coupled with a progressively sedentary lifestyle.^2^ Consequentially, physical inactivity has been considered one of the major public health problems of the twenty-first century,^2^ demonstrated by evidence of an ongoing reduction in physical activity (PA) and fitness in children and adolescents.^3,4^ Thus, the reduction of physical fitness (PF) is associated with an increasing number of health problems, particularly obesity.^5-7^ It is crucial to highlight the significant physiological and psychological changes that children and adolescents undergo, as the result of unhealthy habits and poor lifestyle choices which are established during this period in life and which contribute detrimentally to general health in adulthood.^8^

PF is an integrated measurement of all the functions and structures involved in performing PA. These functions include musculoskeletal, cardiorespiratory, circulatory, metabolic and neurological aspects. Today, the PF is considered one of the most important determinants of the health status in children and adolescents, as well as being a significant predictor of morbidity and mortality in adults together with smoking status and alcohol.^9,10^

The PF is partially genetically determined but may also be strongly influenced by environmental factors, including exercise.^8^ PF-related health criteria includes cardiorespiratory capacity (CRC), strength, flexibility and body composition (particularly adiposity), with the addition of speed and agility in children.^8^

Multiple studies^9-17^ have previously noted that improvements in PF were correlated to a better academic performance (AP). A recent systematic review^13^ on PA, fitness, cognitive function, and academic achievement in children^11^ found evidence to suggest that there are positive associations among PA, fitness, cognition and academic achievement. Nevertheless, the findings are inconsistent and many questions remain regarding how to best incorporate PA within schools. However, the literature suggests no indication that increases in PA negatively affect cognition or academic achievement and PA is important for growth and development and general health. In preadolescent children, fewer studies have reported a positive association between PF and AP,^18-21^ however, no conclusive specific association between domains of PF and AP has been determined.

Previous studies^21^ have reported that the PF of Spanish adolescents must be improved to help protect against cardiovascular disease in adulthood and the fitness level of Spanish adolescents is worse than that of adolescents in other countries. One in 5 Spanish adolescents are estimated to have a level of PF indicative of their future cardiovascular risk. A study in Spain including schoolchildren aged 9-11 years demonstrated that academic achievement scores were positively related to fitness levels.^22^ Therefore, the objective of this study is to examine the relationship between PF, PA, obesity and AP in primary school children in the south of Spain.

## Materials and Methods

### Study design and recruitment

A cross-sectional design study was conducted in primary schools in the province of Córdoba, Spain, in which students aged 9 to 12 years were included. Data was collected from 5 randomly selected schools during the months of April to June 2014. Amongst these, students failing to correctly perform the specified tests were excluded from the sample; students with physical or psychological disabilities, as well as those who had been injured for more than 1 month in the current academic year were excluded. The study included 96 primary school students, all of the included schools were in the urban area of the city of Cordoba and were state schools in a predominantly middle class area. All the schools were in homogenous zones in terms of standards of living (socioeconomic status), with a medium gross income of 23.647 00 € and an average disposable income per capita of households of 19.416 00 €.

In the end, 91 students completed the study and 5 students (5.2%) did not complete the test, due to either change in residence or sports injuries during the test period. An agreement was signed by the school board, Diputación de Cordoba; they were informed about the study and agreed to participate alongside the parents and students.

### Measure

Physical examination

The medical history of the participants was obtained by paediatricians at the University Hospital Reina Sofía of Córdoba, and included information such as personal and family history, the existence of previous disease, long periods of inactivity or any previous medical treatments. A thorough physical examination and an assessment of pre-pubertal development according to Tanner^23^ were performed. According to the tests, results students with physical and psychological disabilities and students with previous recent injuries that limited the capability to perform the physical tests were not included in the study. Anthropometric assessment such as heart rate (HR), blood pressure, weight, height, waist circumference, body mass index (BMI) was conducted as well. Weight and height were measured using a digital scale and stadiometer (Seca 763 Digital Medical Scales with BMI, Center Aisle LLC, United States). Body weight was measured without shoes and in light clothing on a standing scale and body height was measured without shoes. Basal metabolic rate, and percentages of lean mass and body fat was determined using the Tanita BC-418 Segmental Body Composition Analyzer. Obesity is most commonly classified using BMI or by expressing fatness as a percentage of total mass. These single measures of body composition have been shown to be unsatisfactory therefore we used a combination of BMI, waist circumference, percentage of fat mass, lean body mass, percentage of lean mass and basal metabolism.

### Physical fitness assessment 

As part of the assessment of PF, various physical exercises were implemented. Initially, a 10-minute general warm-up encompassing joint mobility, running and activities involving rapid changes of pace and direction were performed. For the evaluation of PF, we included several tests integrated within the EUROFIT,^24^ which are validated and standardised by the Council of Europe. Prior to starting the study, the researchers involved in the EUROFIT tests had 5 training sessions to guarantee validation, reliability and standardization of measurements. The battery of tests were applied in the following order:

(1) Standing Long Jump Test (Broad Jump) with the purpose to evaluate lower limb explosive-strength. A tape measure is used to measure the maximum horizontal distance jumped. The student stands behind a line marked on the ground with feet together. A 2-foot take-off and landing is used, with swinging of the arms and bending of the knees to provide forward drive. The subject attempts to jump as far as possible, landing on both feet without falling backwards. Two attempts allowed.

(2) Eurofit Sit Up Test in 30 seconds. This test measures the endurance of the abdominal and hip-flexor muscles. The goal is to perform as many sit-ups as in 30 seconds on a flat ground with a stopwatch and a partner assisting by anchoring the feet to the ground. On the command ‘Go’, raise the chest so that the upper body is vertical, then return to the floor. Continue for 30 seconds. For each sit up the back must return to touch the floor. The completion of one complete curl up (up and back) counts as one. The sit up must be performed correctly for it to be counted. For the tempo tests, the test is continued until the subject cannot maintain the rhythm or has reached the target number for the test. The maximum number of correctly performed sit ups in 30 seconds is recorded.

(3) Handgrip Strength Test. The purpose of the test is to measure the maximum isometric strength of the hand and forearm muscles. By using a digital precision dynamometer, model EH101 CAMRY, maximum strength in both hands manual pressure is assessed. The subject holds the dynamometer in the hand to be tested, with the arm at right angles and the elbow by the side of the body. When ready the subject squeezes the dynamometer with maximum isometric effort, which is maintained for about 5 seconds. No other body movement is allowed. The subject should be strongly encouraged to give a maximum effort. The best result from several trials for each hand is recorded, with at least 15 seconds recovery between each effort.

(4) Twenty meters endurance Course Navette. The endurance ‘Course-Navette test’ is used to measure maximal aerobic capacity from a test of maximal-incremental-indirect field round of 20 m, using the equations proposed by Léger,^25^ in order to estimate the maximum oxygen consumption (VO2max). The test is performed with continuous running between 2 lines 20 m apart in time to recorded beeps. The test subjects stand behind one of the lines facing the second line, and begin running when instructed by the instructor. The student continues running between the 2 lines, turning when signalled by the recorded beeps. After about one minute, a sound indicates an increase in speed and the beeps will be closer together. This continues each minute (level). If the line is not reached in time for each beep, the subject must run to the line turn and try to catch up with the pace within 2 more ‘beeps’. Also, if the line is reached before the beep sounds, the subject must wait until the beep sounds. The test is stopped if the subject fails to reach the line (within 2 m) for 2 consecutive ends. The reliability and validity of the test to predict the VO2max in children and adolescents have been previously demonstrated.^26^

Finally, a modified version of questionnaire validated by the National Institute of Child Health and Human Development^27^ was carried out. This questionnaire analysed the general PA each child encountered on a daily basis. Examples included mode of transport to school, and duration of participation in physical school programs. A crucial section of the questionnaire also recorded the time dedicated to activities such as watching television, going for walks, help with household chores, and involvement in sports. The questionnaire was filled out by all the students, had a total of 10 questions. Responders agree a value from 1 to 5 for each of the 10 items. The answers for each item start from the lowest activity response and progress to the highest activity response. Once you have a value from 1 to 5 for each of the 10 items, you simply take the mean of these 10 items, which results in the final activity summary score. A total score of 2 or less defined low PF and a total score of 3 or more defined high PF. The questionnaire is appropriate for elementary school-aged children (grades 4-8; approximately ages 8-14). It was administered in the classroom setting and estimated completion time was 20 minutes. This structured alternative format is designed to minimize socially desirable responses and ensure internal consistency and reliability.

### Academic performance evaluation

For the assessment of AP, scores were obtained from the second quarter of the academic year 2013-2014: from April to June 2014, the calculation of AP was based on the total average of scores of basic subjects (math and language) and other subjects, including physical education. Each child was evaluated in exactly the same way. On the other hand, cognitive performance (CP) (non-verbal and verbal ability, abstract reasoning, spatial ability, verbal reasoning and numerical ability) was assessed by the Spanish Overall and Factorial Intelligence Test. A scoring system that has been previously validated by the local educational system (Junta de Andalucía) was used. The students were scored by local experience teachers as 0 - insufficient, 1 - sufficient, 2 - good, 3 - remarkable and 4 - outstanding. There were no significant differences between the schools with respect to the average grade point average (*P* > 0.05).

### Statistical analysis

This is a cross-sectional study. In this subgroup, a significance level of α = 0.05 was established. Participants that did not complete all necessary activities and follow-up was not included in the final results. The normal distribution of the data was measured by the Shapiro-Wilks test. Homogeneity of variances were estimated using the Levene test. The comparison of means between groups of continuous variables with normal distribution was performed by *t* test and for those without normal distribution the Mann–Whitney U test was used, to compare proportions between groups chi-square test was used; and for the association between quantitative variables bivariate Pearson correlation test was used. The data was analysed using the SPSS version 18.0 for Windows (SPSS Inc., Chicago, IL).

## Results

The study included 96 primary school students that were potentially eligible from 326 students. Ninety-one students completed the study and 5 students (5.2%) did not complete the test, due to either change in residence or sports injuries during the test period.

**Figure 1:**
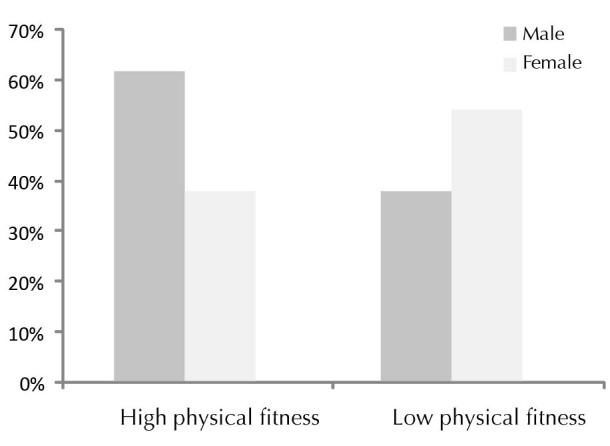
Percentage of physical fitness by gender.

Table 1 summarizes the characteristics of participants by gender as previously described in the EUROFIT. No association of performance with age was found (*P* = 0.512). There were no gender differences in the Course-Navette test (*P* = 0.212), the hand grip dynamometry of the right hand(*P* = 0.566) or left hand (*P* = 0.315) or the standing long jump test (Broad Jump) (*P* = 0.085). An exception was the sit up test in 30 seconds with males scoring higher (*P* = 0.029). Overall no gender differences were detected in the AP (*P* = 0.751) and CP (*P* = 0.470). Table 2 summarizes the main hemodynamic and anthropometric characteristics of children with high or low PF. Differences were found in students with low PF in increased weight (*P* < 0.001), BMI (*P* < 0.001), waist circumference (*P* < 0.001), percentage of fat mass (*P* = 0.001), lean body mass (*P* = 0.001), basal metabolism (*P* = 0.014) and HR (*P* = 0.045). No differences were found in height (*P* = 0.915), systolic (*P* = 0.166) or diastolic (*P* = 0.180) blood pressures. Figure 1 represents the differences in PF according to the gender with the group of students with high PF being made up of 61.5% males and 38.5% females and the group of low PF being made up of 38.5% males and 53.2% females.

Table 3 shows the mean scores for each tests, classified according to the category of PF. Students with better PF obtained better results in the sit up test with an average of 17 (*P* < 0.001). For the maximum aerobic capacity test 4 students managed to complete the distance compared with 1 student in the low PF group (*P* < 0.001), for the standing long jump test (Broad Jump) the high PF group obtained an average of 146 cm (*P* < 0.001) and for the hand grip dynamometer test the high PF group obtained an average 19 kg of force in the right hand and 16 kg of force in the left hand but not statistically difference (*P* = 0.524) and (*P* = 0.386). Finally, Table 4 categorizes the AP according to fitness. Students with better PF showed increased scores in the following subjects: Mathematics (3.10 ± 0.94, *P* = 0.019), Religion (3.61 ± 0.72, *P* = 0.018) and Physical Education (3.69 ± 0.52, *P* < 0.001). Students with better PF performance had significantly higher scores in both the AP and CP. There were no differences between sexes for each of the scores obtained forming the CP. A direct association between maximal aerobic capacity with Mathematics (*r* = 0.325, *P* = 0.02), AP (*r* = 0.349, *P* = 0.001) and CP (*r* = 0.312, *P* = 0.003) was observed. There was also a direct association of better jump tests with higher AP (*r* = 0.328, *P* = 0.002); however, no inverse association between a high BMI was observed with AP (r = -0149, *P* = 0.169) or CP (*r* = -0174, *P* = 0.100). The results of the PF questionnaire indicated that 28.9% of respondent’s watch television for 1 hour a day, 24.4% help with the household work 2 days a week, and 85.7% of students walk to school every day. There was a significant difference between sexes in the level of participation in extra-curricular activities (*P* < 0.001). In specific sports, males (75.9%) participated in football, whilst females (41.7%) participated in basketball. In relation to the practice of PA in a sports club, there were significant differences between sexes (*P* < 0.001) where 81% of boys were in a football club, unlike girls where 34.8% were in a basketball club. Of all the students, those participating in sports clubs showed better results in physical tests (*P* = 0.017).

## Discussion

In recent years, the benefits of the main components of PF in different areas of health have been examined.^9-18^ In relation to obesity, it is observed that children with better cardiorespiratory capacity (CRC) have a lower percentage of general and abdominal adiposity.^27^ Similarly, a healthy cardiovascular profile is associated with the highest levels of CRC and muscle strength in young adults.^28,29^ The fitness of Spanish adolescents is worse than that of other similar countries and it is estimated that 1 in 5 Spanish adolescents have a level of PF that is indicative of future cardiovascular risk. The Spanish adolescents had a worse aerobic capacity than that reported in 11 of the previous 15 studies reported in Netherlands, Belgium, Denmark, Australia, Greece, Sweden, Portugal, Saudi Arabia, Japan, China, and the United States.^22^ Recent studies in our country indicate that when the PF of Spanish teenagers is compared to similar teenagers in other countries, the Spaniards have less muscle strength and worse aerobic capacity, except for American teenagers who were found to have the worst aerobic capacity.^22^ Muscle strength compared to studies in Sweden, Greece and United States showed that Spanish adolescents have less muscle strength and aerobic capacity.

With regards to mental health, most studies have been performed in adults to test the potential beneficial effects of CRC in cognition.^30^ However, studies in young people are scarce. In childhood, it appears that better PF, especially the CRC, is related to an improvement in AP.^31^ In Spain, AP in students is evaluated based on criteria established by the curriculum for primary and secondary education. These are compared with periodical performance tests that are held in different countries, which monitor AP internationally. From the results of such tests, it can be noted that Spain is a country in which there is less influence of socio-economic level on the AP of children. However, it is important to take into account the differences between private and public schools as private schools tend to outperform public schools^19^; this difference becomes insignificant when the socio-economic level of the students is factored in, such that private schools achieve higher AP because students generally come from more advantaged backgrounds, and not because the education centres have better educational systems.^19^ A more recent review using Programme for International Student Assessment (PISA) 2012-Mathematics in 40 countries, including Spain, showed that private school advantage is found only in a handful of countries. Public schools generally perform as well as private subsidized schools and outperform independent schools.^32^

Another body that conducts assessments of AP is the Organization for Economic Co-operation and Development (OECD), which recently published the results of the PISA. PISA is an instrument that assesses the knowledge and skills acquired by students of 15 years of age in maths, literature, and science. The results demonstrate that there is a low proportion of Spanish students attaining high levels of achievement in mathematics, indicating that 25% of Spanish students appear to lack a basic grasp of mathematical competence. As for reading comprehension, the proportion of students achieving levels of excellence is even smaller compared to that of mathematics. In contrast, science achieved the most satisfactory result out of all three subjects.^33^

The American Heart Association has established a relationship between school performance and PF based on the results of the study conducted by Lesley and Cottrell,^34^ suggesting that more focus on PF and physical education at school would result in children being healthier, happier and performing better academically. Studies carried out in several parts of the United States have confirmed this correlation.^18-20,35,36^ A recent study in pubertal children in Murcia, Spain showed that an increase in the number and intensity of physical education sessions have a positive effect on CP (mathematics and language). In addition, the results indicate that improvements in speed, agility and CRC are related to improvements in AP.^37^ According to the latest studies, we can conclude that there are widespread low levels of PF and poor school performance in Spanish children. The results of this study suggest that adequate PF has a positive effect on both CP and AP. In three previous studies involving primary and secondary school students, positive associations were seen between scores of mathematics and PF; aerobic capacity and AP^36^; language and aerobic capacity.^18,19^ In our study, using EUROFIT tests we found some other differences. All physical tests were positively associated with qualifications obtained in mathematics, environmental awareness, religious and physical education. In yet another similar study, conducted in a Californian public school where children were enrolled from fifth, seventh, and ninth grades, the relationship between fitness and achievement appeared to be stronger in females than males^35^; however, in our results, there was no demonstrable difference between genders for the relationship between PF and AP.

In intervention studies of PF to assess AP in adolescents,^37^ the study group with a higher number and intensity of physical education sessions had better AP in the subjects of maths, science and physical education when compared with the control group (2 sessions/week) and a group that underwent four sessions per week. The evaluation tools from this study were replicated in our study. Our results have confirmed these previous findings, in addition to establishing a relationship with the CP as well as one between PF and AP. Ardoy et al^37^ has shown that increasing the number of sessions of physical education alone is insufficient in improving CP or AP, but increasing both intensity and number of sessions appear to have a positive effect. Thus, an increase in the intensity of physical education appears to be a crucial factor in the effect of PF on cognition and academic success. In addition, increased PF levels can reduce the prevalence of childhood obesity and improve AP.^18^ In 4 studies conducted on primary and high school students with validated methodology,^18,19,21,38^ inverse associations of BMI with AP were found, indicating that a lower BMI is positively related to academic success. On the other hand, in 2 studies involving elementary students^39,40^ no such inverse associations of BMI with AP was found. In our study, using other tests, no inverse relationship of BMI with AP or CP was found. The study is limited to the population of 91 primary school students attending 5 schools in the city of Cordoba in the south of Spain. All students were accustomed to having regular, daily physical education classes included in the school program.

## Conclusion

This study confirms previous findings that PF and increased PA is usually positively associated with AP in primary school children; it adds to the growing evidence of the relationship between PF and AP, in particular highlighting the importance of PF and its impact on health. PF should be considered as a useful marker of health throughout childhood and adolescence in addition to improving the quality of life in patients with chronic diseases. This study also emphasizes the importance of PA in school, highlighting it as an ideal intervention against the epidemic of childhood obesity with the added benefits of improving AP. Therefore, improving PF should be considered as a primary objective in promoting public health. Future studies will be aimed at obtaining greater sample size with consideration of other variables (attention span, concentration in class, and intensity of physical education classes) to further illuminate these preliminary results.

## Ethics approval

All participants provided written, informed consent prior to any data collection activities. The Medical Ethics Committee of Hospital Reina Sofia (Cordoba, Spain) approved the study design, study protocols, and the consent procedure.

## Competing interests

The authors are not aware of any affiliations, financial support or memberships that may influence this manuscript; thus, we declare no competing interests.

## Funding

No funding was used to conduct this study or to prepare this manuscript.

## Authors’ contributions

FJFP was the main designer of the study and main author. JVA drafted the manuscript and corresponding author; MVA, FJFO and FJLC performed the data collection; MVA performed statistic analysis and SO native English translation and review.

## Acknowledgments

The authors indicate that no other individuals have contributed to this work.

**Table 1:** Prevalence of characteristics of primary school children in relation to the evaluation tests of fitness and academic performance

**Variables**	**Males** **n = 48**	**Females** **n = 43**	***P*** ^a^
	**Mean±SD**	**Mean±SD**	
Age (y)	11.46±0.50	11.54±0.63	0.512
Broad Jump (cm)	134.01±22.99	124.86±26.17	0.085
Sit up test (n)	16.43±5.61	13.75±5.60	0.029
HGT D Right (kg)	19.02±4.22	18.51±4.21	0.566
HGT D Left (kg)	18.57±11.90	16.65±3.92	0.315
Course-Navette test	2.96±1.72	2.53±1.41	0.212
AP (score)	2.94±0.90	3.00±0.93	0.751
CP (score)	2.72±1.09	2.89±1.08	0.470

Abbreviations: HGT, handgrip strength test, D right: dynamometry of the right hand; D Left, dynamometry of the left hand; AP, academic performance; CP, cognitive performance; SD, standard deviation. ^a^Students *t* test. *P* < 0.05 was considered statistically significant. ^b^Sit up test in 30 seconds.

**Table 2:** Hemodynamic and anthropometric characteristics of primary school children with high and low physical fitness

**Variables**	**High physical fitness** ** n = 39**	**Low physical fitness** ** n = 47**	***P*** ^a^
	**Mean±SD**	**Mean±SD**	
Weight (kg)	21.02±5.62	28.50±8.72	<0.001
Height (cm)	148.39±8.32	148.21±7.11	0.915
BMI (kg/m^2^)	18.92±2.29	22.58±4.05	<0.001
WC (cm)	68.37±6.42	77.12±9.96	<0.001
PFM (%)	41.94±7.63	49.03±10.66	0.001
LBM (%)	34.51±2.46	32.39±3.28	0.001
Basal M (kcal/d)	1317.59±108.36	1387.68±142.67	0.014
SBP (mm Hg)	116.49±10.94	119.83±11.13	0.166
DBP (mm Hg)	65.21±8.99	68.26±11.46	0.180
HR (beats/min)	79.08±10.72	85.28±16.25	0.045

Abbreviations: BMI, body mass index; WC, waist circumference; PFM, percentage of fat mass; LBM, lean body mass: Percentage of lean mass; Basal M, Basal metabolism; SBP, systolic blood pressure; DBP, diastolic blood pressure; HR, heart rate; SD, standard deviation. ^a^Students *t* test. *P* < 0.05 was considered statistically significant.

**Table 3:** Prevalence of Characteristics of Eurofit battery tests of primary school children with high and low physical fitness

**Variables**	**High physical fitness** ** n = 39**	**Low physical fitness** ** n = 47**	***P***
	**Mean±SD**	**Mean±SD**	
Broad Jump (cm)	146.23±16.93	117.15±22.48	<0.001
Sit up test^b^ (n)	17.95±3.94	13.17±6.05	<0.001
HGT D Right (kg)	19.17±3.80	18.59±4.52	0.524
HGT D Left (kg)	16.81±4.54	18.56±11.83	0.386
Course-Navette test	4.18±1.25	1.57±0.50	<0.001

Abbreviations: HGT, handgrip strength test, D right: dynamometry of the right hand; D Left, dynamometry of the left hand; SD, standard deviation. ^a^Students *t* test. *P* < 0.05 was considered statistically significant. ^b^Sit up test in 30 seconds.

**Table 4:** Mean achievement in academic performance according to fitness category

**Variables**	**High physical fitness** ** n = 39**	**Low physical fitness** ** n = 47**	***P***
	**Mean±SD**	**Mean±SD**	
Medium (score)	3.21±0.97	2.66±1.18	0.024
Artistic (score)	3.10±0.75	2.79±1.04	0.118
Physical education (score)	3.69±0.52	3.13±0.79	<0.001
Language (score)	3.10±0.94	2.68±1.23	0.084
English (score)	2.87±1.43	2.38±1.48	0.126
Mathematics (score)	3.10±0.94	2.53±1.21	0.019
Religion (score)	3.61±0.72	3.09±1.13	0.018
Citizenship (score)	3.00±1.07	2.85±1.19	0.653
AP (score)	3.31±0.67	2.73±0.98	0.004
CP (score)	3.10±0.87	2.60±1.17	0.032

Abbreviations: Medium, Environmental knowledge; Artistic, art education; Citizenship, citizenship education; AP, academic performance; CP, cognitive performance; SD, standard deviation. ^a^Students *t* test. *P* < 0.05 was considered statistically significant.
